# PRMT2 promotes RCC tumorigenesis and metastasis via enhancing WNT5A transcriptional expression

**DOI:** 10.1038/s41419-023-05837-6

**Published:** 2023-05-12

**Authors:** Zhongwei Li, Chaozhen Chen, Hongmei Yong, Lei Jiang, Pengfei Wang, Sen Meng, Sufang Chu, Zhen Li, Qingxiang Guo, Junnian Zheng, Jin Bai, Hailong Li

**Affiliations:** 1 Cancer Institute, Xuzhou Medical University, Xuzhou, Jiangsu China; 2 Center of Clinical Oncology, The Affiliated Hospital of Xuzhou Medical University, Xuzhou, Jiangsu China; 3 Jiangsu Center for the Collaboration and Innovation of Cancer Biotherapy, Cancer Institute, Xuzhou Medical University, Xuzhou, Jiangsu China; 4 Department of Urology, The Affiliated Hospital of Xuzhou Medical University, Xuzhou, Jiangsu China; 5 Department of Oncology, The Affiliated Huai’an Hospital of Xuzhou Medical University and The Second People’s Hospital of Huai’an, Huaian, Jiangsu China

**Keywords:** Renal cell carcinoma, Histone post-translational modifications, Methylation, Oncogenes, Targeted therapies

## Abstract

Protein arginine methyltransferase 2 (PRMT2) is involved in several biological processes via histone methylation and transcriptional regulation. Although PRMT2 has been reported to affect breast cancer and glioblastoma progression, its role in renal cell cancer (RCC) remains unclear. Here, we found that PRMT2 was upregulated in primary RCC and RCC cell lines. We demonstrated that PRMT2 overexpression promoted RCC cell proliferation and motility both in vitro and in vivo. Moreover, we revealed that PRMT2-mediated H3R8 asymmetric dimethylation (H3R8me2a) was enriched in the WNT5A promoter region and enhanced WNT5A transcriptional expression, leading to activation of Wnt signaling and malignant progression of RCC. Finally, we confirmed that high PRMT2 and WNT5A expression was strongly correlated with poor clinicopathological characteristics and poor overall survival in RCC patient tissues. Our findings indicate that PRMT2 and WNT5A may be promising predictive diagnostic biomarkers for RCC metastasis. Our study also suggests that PRMT2 is a novel therapeutic target in patients with RCC.

## Introduction

Renal cell carcinoma (RCC) is the third most common urological cancer with an increasing incidence [1]. As one of the most treatment-resistant tumors, there is no effective treatment for RCC after metastasis [2]. Consequently, new indicators for evaluating the probability of metastasis and effective therapeutic targets for therapeutic options are urgently needed in patients with metastatic RCC.

Protein arginine methyltransferases (PRMTs) catalyze arginine methylation of histones and nonhistone proteins [3, 4]. PRMTs family members (PRMT1–9) can result in three types of methylarginines: monomethylarginine (MMA), asymmetric dimethylarginine (ADMA), and symmetric dimethylarginine (SDMA). The PRMTs family members are sorted to three types according to the type of methylarginines: type I (PRMT1, PRMT2, PRMT3, PRMT4, PRMT6, and PRMT8) mediating MMA and ADMA modification, type II (PRMT5 and PRMT9) mediating MMA and SDMA; type III (PRMT7) only mediating MMA modification. Previous studies have demonstrated that arginine methylation of histones regulates gene transcription and H4 histone stability [5–7]. Several studies have reported that arginine methylation of proteins plays a crucial role in the progression of malignant cancer [8, 9]. For instance, we reported that PRMT1-mediated EZH2–R342 dimethylation facilitates breast cancer tumorigenesis and metastasis [10–12]. Recent studies have shown that protein arginine methyltransferase 2 (PRMT2), a member of the PRMT family, is responsible for catalyzing H3R8 asymmetric dimethylation (H3R8me2a), which activates PRMT2 target gene expression [13]. Although several reports have shown that PRMT2 expression is correlated with breast cancer and glioblastoma (GBM) progression [14], there is no report on PRMT2 function in RCC or other types of cancers. Therefore, we wondered whether PRMT2 affects the malignant progression of RCC.

Wnt signaling, a classic signaling pathway, is usually hyperactivated in many cancers [15] and regulates developmental, cellular, and pathogenic processes, including embryogenesis [16], stem cell maintenance [17], and oncogenesis [18]. The binding of Wnt family members or their receptors is considered to be the beginning of Wnt signaling stimulation [19]. Wnt family member 5A (WNT5A), an important Wnt protein family member, can strongly activate the Wnt signaling pathway by binding to the FZD4-LPR5 complex receptor and then accumulating β-Catenin, which contributes to poor outcomes in cancer patients [20]. At present, Wnt signaling has been confirmed to be overactivated in RCC progression [21], but the detailed mechanism of how the Wnt signaling pathway is activated in RCC cells has not yet been reported.

In this study, we showed that PRMT2 promotes RCC cell proliferation and motility in vitro and in vivo. We illustrated that PRMT2 promotes WNT5A expression by mediating the enrichment of H3R8me2a modification on the WNT5A promoter and activates Wnt signaling. Moreover, we confirmed that WNT5A is required for PRMT2 to enhance RCC malignancy. Finally, we demonstrated positive correlations between PRMT2 and WNT5A expression and poor clinicopathological characteristics, and poor survival in RCC patients, indicating that targeting PRMT2 is a potential therapeutic strategy in RCC patients, especially for metastatic RCC patients.

## Materials and methods

### Bioinformatics analysis

The TCGA database (https://www.cancer.gov/tcga) was used to analyze the standardized expression levels of PRMT2 mRNA in kidney renal cell carcinoma. The University of Alabama at the Birmingham Cancer Data Analysis Portal (http://ualcan.path.uab.edu) provides PRMT2 expression in RCC analysis options using data from the Clinical Proteomic Tumor Analysis Consortium (CPTAC).

### Cell culture and cell treatment

HEK293T, HK-2, ACHN, 786-O, Caki-1, KETR3, and OS-RC2 cell lines were obtained from American Type Culture Collection (ATCC, Manassas, VA, USA). 786-O, Caki-1, KETR3, and OS-RC2 cells were cultured in RPMI-1640 medium (KGM31800NH-500, KeyGEN BioTECH) supplemented with 10% fetal bovine serum, 100 U/ml penicillin, and 100 μg/ml streptomycin and incubated in a 37^◦^C humidified incubator with 5% CO_2_. ACHN cells were cultured in MEM (KGM41500N-500, KeyGEN BioTECH), HEK293T, and HK-2 cells were cultured in DMEM (KGM12800NH-500, KeyGEN BioTECH), and the other cell culture conditions were the same as those described above.

### Plasmids and transfections

Plasmids containing short hairpin RNA against human PRMT2 and negative control were purchased from GenePharma Technology (Shanghai, China). The WNT5A plasmid was purchased from MiaoLingPlasmid (Wuhan, China, #P38107). myc-PRMT2 of human and empty vectors were kindly provided by Professor Wuhan Xiao (Institute of Hydrobiology, Chinese Academy of Sciences). All plasmids were transfected into RCC cells using Lipofectamine 2000, according to the manufacturer’s instructions. The shPRMT2 sequences are shown in the Supplementary Information.

### RNA extract, reverse transcription, and qRT-PCR

RNA extraction, reverse transcription, and qRT-PCR were performed as previously described [22]. All primers were obtained from Genewiz (Suzhou, China). Primer sequences are listed in the Supplementary Information.

### Western blot analysis and antibodies

Western blot analysis was performed as previously described [23]. Specific primary antibodies against PRMT2 (66885-1-Ig, Proteintech), Cyclin-B1 (12231P, Cell Signaling Technology), Cyclin-D1 (60186-1-Ig, Proteintech), GAPDH (60004-1-AP, Proteintech), myc-Tag (60003-2-Ig, Proteintech), FN1 (15613-1-AP, Proteintech), E-cadherin (20874-1-AP, Proteintech), N-cadherin (22018-1-AP, Proteintech), Snail-1 (22018-1-AP, Proteintech), WNT5A (ab229200, Abcam), β-Catenin (51067-2-AP, Proteintech), c-Myc (10828-1-AP, Proteintech), H3R8me2a (39651, Active motif) were used for western blot assays, all the western blot experiments were repeated at least three times.

### Cell proliferation, migration, and invasion assays

Cell proliferation, migration, and invasion assays were performed according to the previous report [24]. The detailed protocols were shown in Supplementary Information.

### Cell cycle assay

Cell cycle assay was performed according to the previous report [25]. The detailed protocol is shown in Supplementary Information.

### RNA sequencing

ACHN cells were transfected with shRNA targeting human PRMT2 or the negative control for 48 h. Some cells were used to assess transfection efficiency by western blotting, and other cells were collected using the Trizol reagent. Total RNA samples were sequenced using Illumina HiSeq2000 (Majorbio Technology Inc., Shanghai, China). Significance analysis of RNA-seq data was used to identify genes that were significantly up or downregulated by treatment using unlogged data, with a false discovery rate (FDR) of less than 0.05. Fold-change (FC) was computed using average transcript levels compared to the negative control values, which were log2-transformed and computed for Spearman’s correlation coefficients between samples. SAM analysis was used to calculate differential gene expression.

### Luciferase reporter assay

The experiments were performed in accordance with a previous report [26]. A pGL3.0-WNT5A (−1000/+0) reporter gene plasmid was constructed using GeneWiz (Suzhou, China). The detailed protocol and WNT5A promoter DNA sequences (−1000/+0) are shown in Supplementary Information.

### Chromatin immunoprecipitation (ChIP)

Chromatin immunoprecipitation (ChIP) assays were performed using a ChIP Assay Kit (Beyotime, Cat#P2078), according to a previous report [27]. The primers used to amplify the target gene promoters are shown in the Supplementary Information.

### Stable cell lines generation

HEK293T cells were transfected with short hairpin RNAs against human PRMT2 or the corresponding negative control which was purchased from GenePharma Technology (Shanghai, China) using Lipofectamine 2000. Virus-containing supernatant was harvested 48 h post-transfection. ACHN cell line was infected with the virus to create stable cell lines, and purinomycin was used for screening 48 h after infection. Specific methods as detailed in our previous study [11].

### Animal works of tumor xenograft model and lung-colonization metastasis model

Animal experiments were approved by the Animal Care Committee of Xuzhou Medical University, Xuzhou, China. Male BALB/c nude mice (6–8 weeks old) were obtained from GemPharmatech. The tumor xenograft model and lung colonization metastasis model were established as previously described [26].

### Immunohistochemistry (IHC) staining

IHC assays were conducted following the standard streptavidin-peroxidase method, as previously reported [28]. The primary antibodies used were as follows: anti-PRMT2 1:100 dilution, anti-WNT5A 1:100 dilution, anti-Ki-67 1:100 dilution, and anti-Cyclin-D1 1:100 dilution. The detailed IHC assessment method is described in the Supplementary Information.

### Patients and sample collection

Renal cancer tissue microarray (TMA) slides and corresponding patients’ clinicopathological information were obtained according to a previous report [28]. The detailed patient’s clinicopathological information was shown in Supplementary Information.

### Immunohistochemistry of TMA assays and evaluation of the immune-staining method

TMA immunohistochemistry and immunostaining were performed as previously described [28]. Detailed protocols were provided in the Supplementary Information.

### Statistical analysis

All data are presented as the mean ± SD in at least three biological experiments. Statistical analyses of TMA slide staining were performed using the SPSS 20 software. Student’s *t*-test was used to analyze the statistical significance of differences, and statistical significance was set at *P* < 0.05. Statistical analyses were performed using GraphPad Prism software.

## Results

### PRMT2 is highly expressed in RCC patients and RCC cell lines

**Fig. 1:**
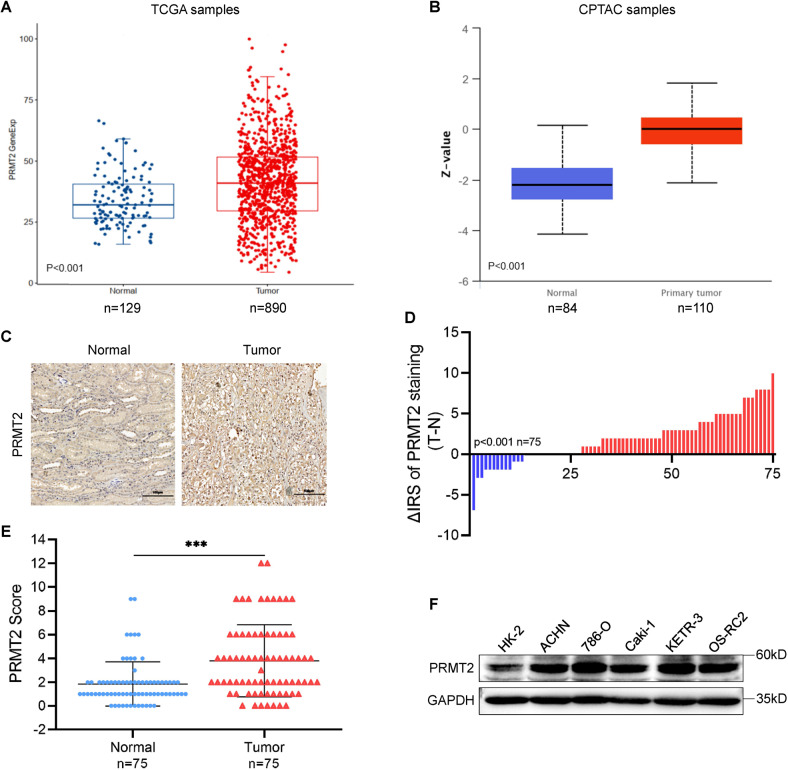
PRMT2 is highly expressed in RCC patients and RCC cell lines. **A** The mRNA expression of PRMT2 was analyzed in pan-RCC (kidney renal clear cell carcinoma, kidney chromophobe, kidney renal papillary cell carcinoma) according to the TCGA datasets. The significance level was determined using one-way ANOVA followed by Dunnett’s multiple comparisons tests. **B** The protein expression of PRMT2 was analyzed in RCC according to the TCGA datasets on the University of Alabama at Birmingham cancer data analysis portal (UALCAN). **C** Representative images of PRMT2 IHC staining in 75 pairs of cancer tissue and cancer-adjacent normal tissues from patients with RCC. **D**, **E** Significance of IHC staining scores were tested by paired-samples *t*-test and Wilcox matched-pairs test. **F** The protein expression of PRMT2 in a human renal tubular epithelial cell line (HK-2) and RCC cell lines (ACHN, 786-O, Caki-1, KETR-3, and OS-RC2) were assessed by western blots.

To explore the role of PRMT2 in RCC, The Cancer Genome Atlas (TCGA) database was used to analyze PRMT2 expression levels in RCC patients. The results showed that the mRNA expression of PRMT2 significantly increased in primary RCC tumors (Fig. 1A). We also used the Clinical Proteomic Tumor Analysis Consortium database (CPTAC) to determine the protein level of PRMT2 in patients with clear cell RCC. The data showed that PRMT2 expression was drastically increased in clear cell RCC tissues compared with normal tissues (Fig. 1B).

We further assessed PRMT2 expression in cancerous tissues and paired normal adjacent tissues from 75 patients with RCC using IHC assays. IHC staining showed that the expression of PRMT2 was strongly increased in RCC tumor tissues compared to normal tissues (Fig. 1C–E). In addition, we assessed PRMT2 expression in different renal cell carcinoma cell lines using western blotting. We found that PRMT2 expression was dramatically upregulated in the RCC cell lines, especially in the 786-O and ACHN cell lines, compared to that in the human kidney-2 (HK-2) cell line (Fig. 1F). Taken together, these data demonstrate that PRMT2 is highly expressed in RCC tissues and cell lines. This strongly suggests that PRMT2 plays a key role in RCC progression.

### PRMT2 promotes RCC proliferation and migration in vitro

**Fig. 2:**
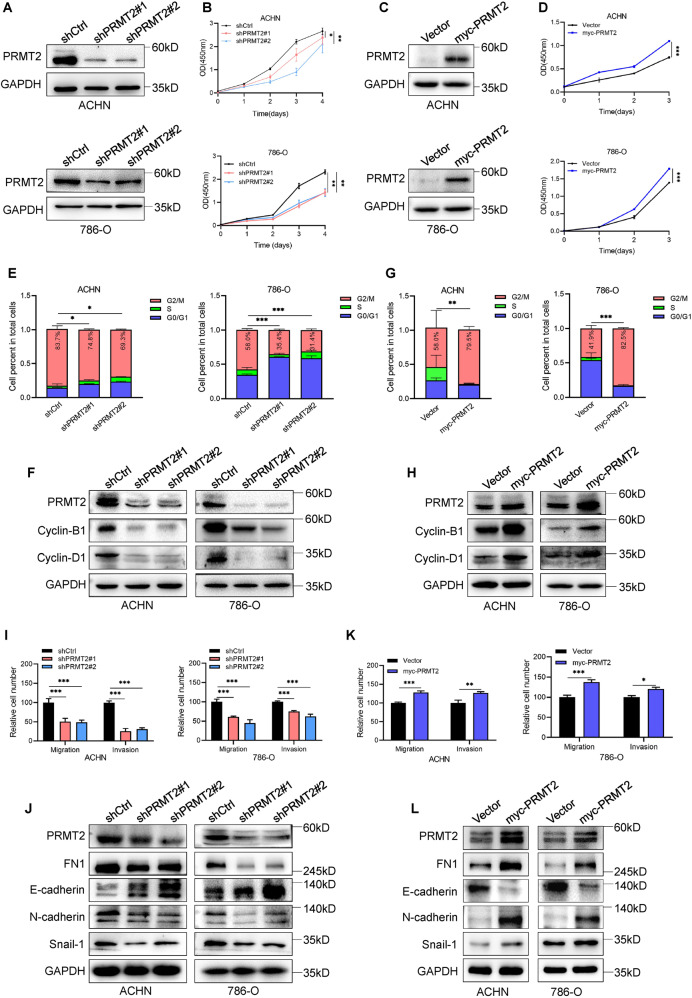
PRMT2 promotes RCC cell proliferation and migration. **A**–**D** PRMT2 expression was assessed by western blots in ACHN and 786-O cell lines after knockdown (**A**) or overexpression (**C**) of PRMT2, followed by CCK-8 assays (**B**, **D**). **E**–**H** Flow cytometry was used to detect and quantify cell cycle arrest in ACHN and 786-O cell lines after knockdown (**E**) or overexpression (**G**) of PRMT2, and cell cycle-related proteins were assessed by western blots (**F**, **H**). **I**–**L** Detection of RCC cell migration by Transwell assays in ACHN and 786-O cells after knockdown (**I**) or overexpression (**K**) of PRMT2. Biomarkers of EMT were assessed by western blots after knockdown (**J**) or overexpression (**L**) of PRMT2. Data are presented as the mean ± SD. *P < 0.05; **P < 0.01; ***P < 0.001.

To identify the function of PRMT2 in RCC, we knocked down PRMT2 expression in the ACHN and 786-O cell lines using two different short hairpin RNAs (shRNAs). We first confirmed that PRMT2 was significantly decreased at both the mRNA and protein levels (Fig. 2A and Fig. S1A). Knockdown of PRMT2 led to decreased proliferation of ACHN and 786-O cells, as determined by Cell Counting Kit-8 (CCK-8) assays (Fig. 2B). In contrast, our CCK-8 assays also revealed that the proliferation ability of ACHN and 786-O cells increased after ectopic expression of PRMT2 (Fig. 2C, D). Besides, we also confirmed that overexpression of PRMT2 promoted HK-2 cell proliferation ability (Fig. S2A, B).

Subsequently, flow cytometry assays were used to analyze cell cycle progression and apoptosis. We found that the percentage of the G2/M phase was decreased after the knockdown of PRMT2 in ACHN and 786-O cells. The results showed that silencing PRMT2 mainly led to ACHN and 786-O cell cycle arrest in S to G2/M phase (Fig. 2E). Furthermore, western blot assays were used to analyze the expression of cell cycle-related proteins. Our data showed that PRMT2 depletion inhibited cyclin-B1 and cyclin-D1 expression (Fig. 2F). Conversely, the cell cycle accelerated when PRMT2 was overexpressed in ACHN and 786-O cells (Fig. 2G). Correspondingly, cyclin-B1 and cyclin-D1 levels increased after PRMT2 overexpression in ACHN and 786-O cells (Fig. 2H). These data clearly indicate that PRMT2 facilitates RCC cell proliferation in vitro.

Cancer cells have unique mechanical properties that result in metastasis, which is tightly associated with epithelial-mesenchymal transition (EMT) progression [29, 30]. Transwell assays were performed to determine the role of PRMT2 in the motility of RCC cells. The results showed that PRMT2 deficiency restrained the migration and invasion of ACHN and 786-O cells (Fig. 2I). Consistently, our immunoblot assays confirmed that the mesenchymal biomarkers (FN1, N-cadherin, and Snail-1) were decreased, whereas the epithelial biomarker E-cadherin was increased when PRMT2 was knocked down in ACHN and 786-O cells (Fig. 2J). In contrast, our Transwell assays also showed that PRMT2 overexpression enhanced the migration and invasion abilities of ACHN, 786-O, and HK-2 cells (Fig. 2K and Fig. S2C). The above-mentioned EMT markers showed the opposite changes when PRMT2 was overexpressed in ACHN and 786-O cells compared to when PRMT2 was silenced (Fig. 2L). In conclusion, these data strongly suggest that PRMT2 promotes RCC cell invasion and migration in vitro.

### PRMT2 stimulates Wnt signaling by activating WNT5A transcriptional expression

**Fig. 3:**
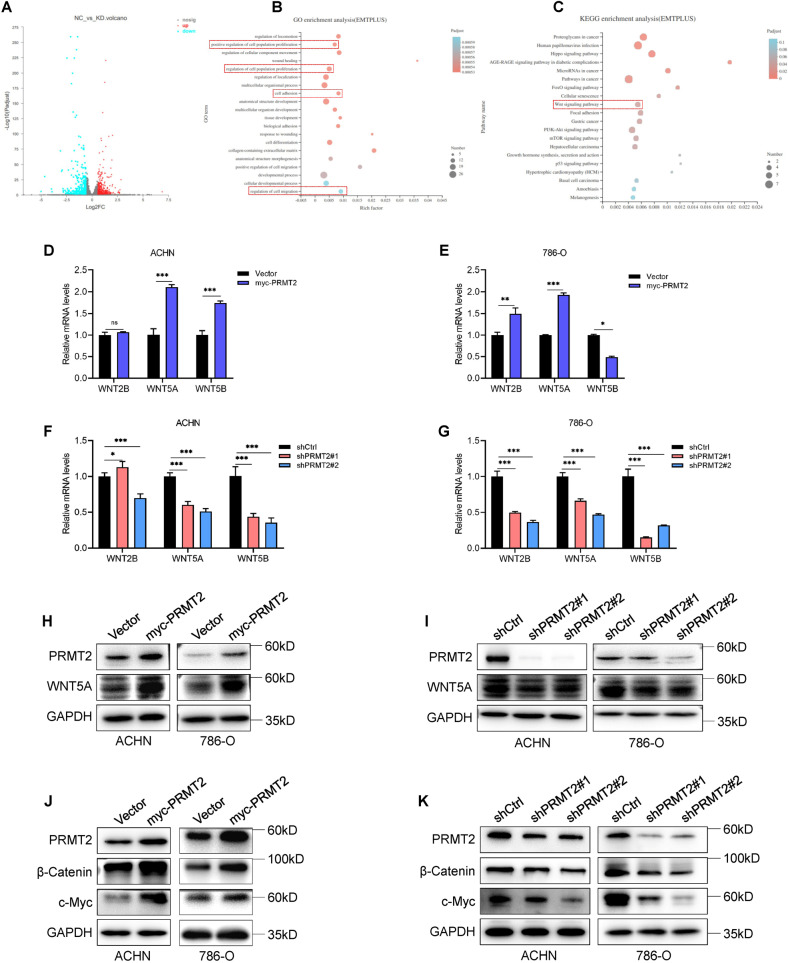
PRMT2 increases WNT5A transcriptional expression and activates Wnt signaling. **A** Volcano plot showed the differential transcripts expression by RNA-seq analysis in the ACHN-shCtrl group and the ACHN-shPRMT2 group. (Upregulated genes number: 404, whereas downregulated genes number: 396). **B** Gene Ontology (GO) enrichment analysis of the gene set by RNA-seq assays. Top functions are shown. **C** Kyoto Encyclopedia of Genes and Genomes (KEGG) enrichment analysis of the gene set by RNA-seq assays. Top signaling is shown. **D**–**G** qRT-PCR assays were used to verify the results of RNA-seq in ACHN and 786-O cells after knockdown or overexpression of PRMT2. **H**, **I** Protein of WNT5A was assessed by western blots in ACHN and 786-O cell lines after overexpression (**H**) or knockdown (**I**) of PRMT2. **J**, **K** The Wnt signaling cascade-related genes (β-Catenin, c-Myc) were assessed by western blots in ACHN and 786-O cell lines after overexpression (**J**) or knockdown (**K**) of PRMT2. Data are presented as the mean ± SD. *P < 0.05; **P < 0.01; ***P < 0.001.

Studies have reported that arginine methylation of histones (such as H4R3, H3R8) regulates its target gene’s transcriptional expression [7, 14]. Therefore, we performed RNA sequencing to determine the changes in PRMT2 target gene expression when PRMT2 was silenced in ACHN cells. Bioinformatics analysis of our RNA sequencing data revealed that 404 genes were upregulated and 396 genes were downregulated after silencing PRMT2 (Fig. 3A). To determine how PRMT2 downregulation alters the transcriptional expression of genes in ACHN cells, the first 200 genes in the downregulated group were used as the defined gene set. Gene Ontology (GO) enrichment analysis demonstrated that the gene set was significantly associated with cell differentiation, cell adhesion, and regulation of locomotion (Fig. 3B). Kyoto Encyclopedia of Genes and Genomes (KEGG) enrichment analysis showed that there were genes in the gene set enriched in the Wnt signaling pathway (Fig. 3C). In fact, our RNA-seq data showed that genes of the WNT family (WNT2B, WNT5A, and WNT5B) were indeed significantly downregulated in the gene set (Supplementary Table 1).

To verify the RNA sequencing results, we performed qRT-PCR to determine the expression levels of WNT2B, WNT5A, and WNT5B in ACHN and 786-O cells. Our results showed that high PRMT2 expression only strongly activated WNT5A but not WNT2B or WNT5B expression at the transcriptional level in ACHN and 786-O cells (Fig. 3D, E). In addition, our data also confirmed that PRMT2 deficiency only significantly led to a reduction in WNT5A expression at the transcriptional level in ACHN and 786-O cells (Fig. 3F, G). Based on the homogeneity of PRMT2 regulating WNT5A expression in ACHN and 786-O cells, we proposed that PRMT2 can activate WNT5A transcription and focused on WNT5A as the main target gene in the subsequent experiments.

Furthermore, western blot data showed that WNT5A expression increased when PRMT2 was overexpressed (Fig. 3H), whereas WNT5A was decreased at the protein level after knockdown of PRMT2 (Fig. 3I). Consistently, we explored the effects of PRMT2 on the Wnt signaling pathway by detecting the expression of key Wnt signaling pathway genes (β-Catenin and c-Myc). We found that PRMT2 overexpression increased β-Catenin and c-Myc expression (Fig. 3J). Similar results were obtained when ectopic PRMT2 expression was observed in HEK293T cells (Fig. S1B). Conversely, PRMT2 deficiency downregulated β-Catenin and c-Myc expression in ACHN and 786-O cells (Fig. 3K). These results indicate that PRMT2 is responsible for the activation of the Wnt signaling pathway by stimulating WNT5A transcriptional expression.

### PRMT2 activates WNT5A transcription by facilitating H3R8me2a enrichment on its promoter

RNA sequencing data indicated that PRMT2 increased WNT5A expression at the transcriptional level. Recent reports have shown that PRMT2 can activate target gene expression by catalyzing histone H3R8 asymmetric dimethylation in these gene promoter regions [13, 14, 31, 32]. We speculated that PRMT2 activation of WNT5A transcription might involve a similar mechanism in RCC cells.

**Fig. 4:**
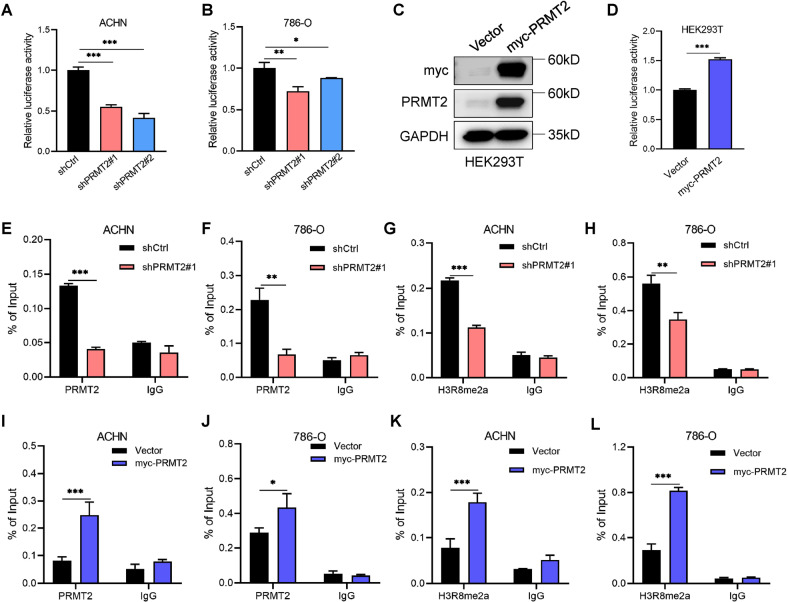
PRMT2 directly activates WNT5A transcription via mediating H3R8me2a enrichment on its promoter. **A**, **B** Luciferase reporter gene assays detecting WNT5A promoter region activity after knockdown PRMT2 in ACHN and 786-O cells. **C**, **D** Luciferase reporter gene assays detecting WNT5A promoter region activity after over-expressing PRMT2 in HEK293T cells. **E**–**H** ChIP assays were undertaken in ACHN and 786-O cells after the knockdown of PRMT2 with antibodies against PRMT2 and H3R8me2a. **I**–**L** ChIP assays were undertaken in ACHN and 786-O cells after the overexpression of PRMT2 with antibodies against PRMT2 and H3R8me2a. IgG was used for negative control. The enrichment of PRMT2 and H3R8me2a binding to the WNT5A promoter was quantified with qRT-PCR. Data are presented as the mean ± SD. *P < 0.05; **P < 0.01; ***P < 0.001.

To verify our hypothesis, we first constructed a luciferase reporter gene bearing the WNT5A promoter region to perform reporter gene assays (Fig. S3A). Luciferase reporter assays showed that WNT5A transcription was downregulated after PRMT2 knockdown (Fig. 4A, B). In contrast, we found that overexpression of PRMT2 activated WNT5A transcription in our luciferase assays (Fig. 4C, D). These results reveal that PRMT2 directly activates WNT5A transcription.

To further determine whether PRMT2 mediates H3R8me2a accumulation on the WNT5A promoter region, we performed ChIP assays to assess the enrichment of PRMT2 and H3R8me2a on the WNT5A promoter. Several pairs of ChIP qRT-PCR primers were designed for the WNT5A promoter region (Fig. S3B). We then selected the highest-enrichment primer on the WNT5A promoter to carry out the following ChIP experiments (Fig. S3C). The ChIP data revealed that PRMT2 knockdown significantly reduced PRMT2 enrichment on the WNT5A promoter region (Fig. 4E, F), confirming that PRMT2 is directly recruited to the WNT5A promoter. Consistently, we also found that the amount of PRMT2-mediated H3R8me2a decreased on the WNT5A promoter region after silencing PRMT2 in RCC cells (Fig. 4G, H). In contrast, overexpression of PRMT2 increased WNT5A promoter enrichment of PRMT2 and H3R8me2a (Fig. 4I–L). Collectively, these findings demonstrate that PRMT2 increases WNT5A expression via PRMT2-mediated enrichment of H3R8me2a on the WNT5A promoter in RCC cells.

### WNT5A is required for PRMT2-mediated RCC cell proliferation and migration

**Fig. 5:**
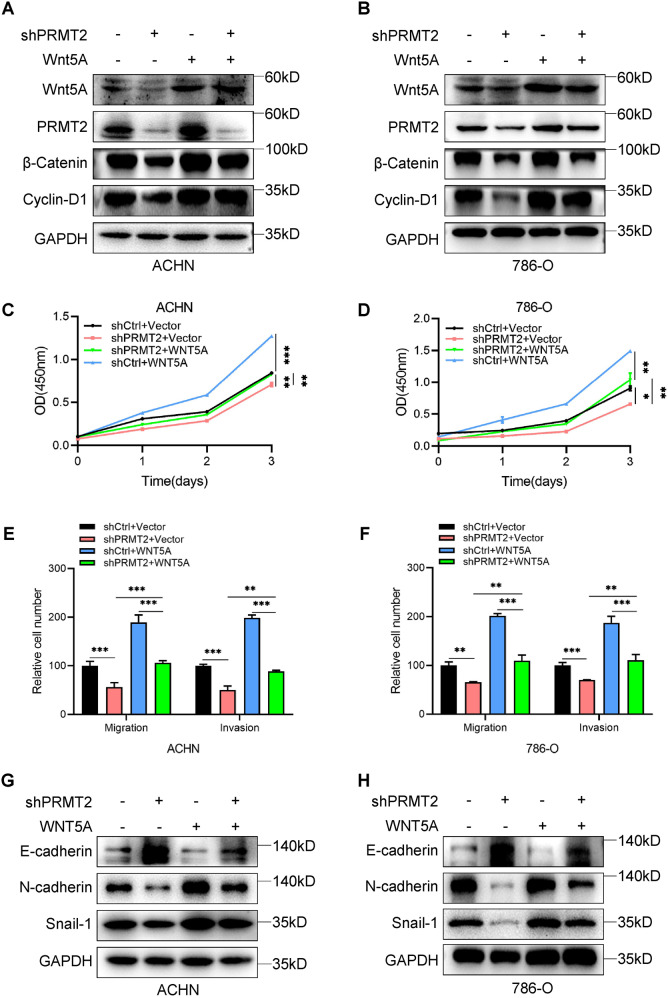
PRMT2 promotes RCC cell proliferation and migration via WNT5A activating Wnt signaling. **A**, **B** The state of Wnt signaling was assessed by western blots in ACHN and 786-O cell lines with overexpression WNT5A after the knockdown of PRMT2. **C**, **D** The proliferation ability of ACHN and 786-O cells was tested by CCK-8 with overexpression of WNT5A after the knockdown of PRMT2. **E**, **F** Detection of RCC cell migration by transwell assays in ACHN and 786-O cells with overexpression of WNT5A after knockdown PRMT2. **G**, **H** The EMT markers were assessed by western blots in ACHN and 786-O cell lines with WNT5A overexpressing after knockdown PRMT2. Data are presented as the mean ± SD. *P < 0.05; **P < 0.01; ***P < 0.001.

To further investigate the role of WNT5A in PRMT2-mediated epigenetic regulation, we performed rescue experiments in ACHN-shPRMT2 and 786-O-shPRMT2 cells by ectopic expression of WNT5A. Western blot assays showed that β-Catenin and cyclin-D1 were increased after overexpression of exogenous WNT5A in PRMT2 knockdown RCC cells (Fig. 5A, B). CCK-8 assays showed that cell proliferation recovered after overexpression of exogenous WNT5A in PRMT2 knockdown RCC cells (Fig. 5C, D). In addition, the proliferation of ACHN and 786-O cells were recovered after treatment with LiCl (a classical agonist of the Wnt signaling pathway) in PRMT2-knockdown RCC cells (Fig. S4A, B). Our data indicate that WNT5A is necessary for PRMT2 to promote RCC cell proliferation.

Moreover, we also detect the role of WNT5A in the PRMT2 enhancement of RCC cell motility. Transwell assays revealed that the migration and invasion abilities of ACHN and 786-O cells increased after overexpression of WNT5A in PRMT2-knockdown RCC cells (Fig. 5E, F). We also investigated the role of WNT5A in the PRMT2 facilitation of the EMT process, and western blot assays showed that Snail-1 and N-cadherin increased while E-cadherin decreased after ectopic expression of WNT5A in PRMT2-knockdown RCC cells (Fig. 5G, H). Besides, the cell motility of ACHN and 786-O cells was increased after treatment with LiCl in PRMT2-knockdown RCC cells (Figure S4C-D). These results suggest that WNT5A is essential for PRMT2-induced RCC cell proliferation and migration.

### PRMT2 promotes RCC tumorigenesis and metastasis in vivo

**Fig. 6:**
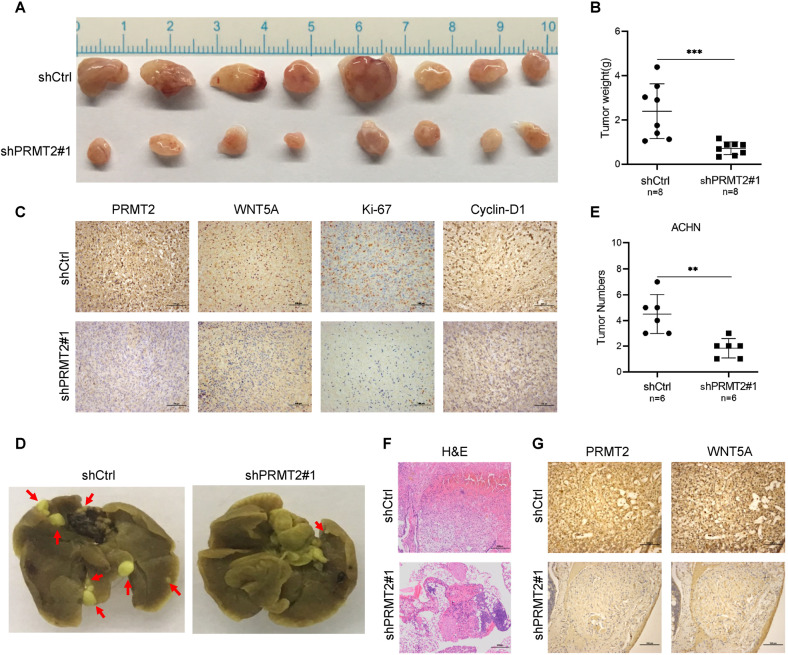
PRMT2 promotes RCC tumorigenesis and metastasis by increasing WNT5A expression in vivo. **A**, **B** ACHN-shCtrl cells or ACHN-shPRMT2 cells were subcutaneously injected into the flanks of BALB/c nude mice. *n* = 8 for each group. The subcutaneous tumors were weighed up. **C** IHC detected PRMT2, WNT5A, Ki-67, and Cyclin-D1 in subcutaneous tumors. **D**, **E** The same number of ACHN-shCtrl cells or ACHN-shPRMT2 cells were injected into BALB/c nude mice through the tail vein. *n* = 6 for each group. Representative lung images were shown when mice were sacrificed after 8 weeks (**D**), the red arrows denote the metastatic nodules, and lung metastatic nodules were examined macroscopically (**E**). **F** Hematoxylin/Eosin-stained (H&E) lung sections were shown. **G** Representative images of the IHC staining of PRMT2 and WNT5A in lung metastasis sections. Data are presented as the mean ± SD. **P < 0.01; ***P < 0.001.

Furthermore, we wanted to determine whether PRMT2 and WNT5A play important roles in RCC cell proliferation and migration in vivo. First, the same number of ACHN-shCtrl or ACHN-shPRMT2 cells was subcutaneously injected into the flanks of BALB/c nude mice for tumorigenesis assays. Four weeks later, the mice were sacrificed, and the subcutaneous tumors were weighed. Our results showed that tumors in the shPRMT2 group were significantly smaller and weighed less than those in the shCtrl group (Fig. 6A, B). IHC staining confirmed that tumors derived from ACHN-shPRMT2 cells had low expression levels of PRMT2 and WNT5A compared to those in the control group (Fig. 6C). The expression levels of Ki-67 and cyclin-D1 were lower in the shPRMT2 group than in the control group (Fig. 6C). These results demonstrate that PRMT2 is necessary for RCC tumorigenesis in vivo.

Subsequently, to investigate the role of PRMT2 in RCC metastatic ability in vivo, the same number of ACHN-shCtrl or ACHN-shPRMT2 cells was injected into BALB/c nude mice through the tail vein. Mice were sacrificed after 8 weeks, and lung metastatic nodules were examined after fixation using Bouin’s method. Our observations revealed that injecting ACHN-shPRMT2 cells resulted in fewer and smaller macroscopic lung metastatic nodules than in the shCtrl group (Fig. 6D, E). IHC staining assays were used to study the function of PRMT2 in the metastatic processes. The results showed that PRMT2 and WNT5A expression was lower in the shPRMT2 group than in the shCtrl group (Fig. 6F, G). These data reveal that PRMT2 is responsible for ACHN metastasis in vivo. Taken together, the above data strongly suggest that PRMT2 also plays a critical role in the tumorigenesis and metastasis of RCC in vivo.

### High PRMT2 and WNT5A expression positively correlates with poor prognosis in RCC patients

**Fig. 7:**
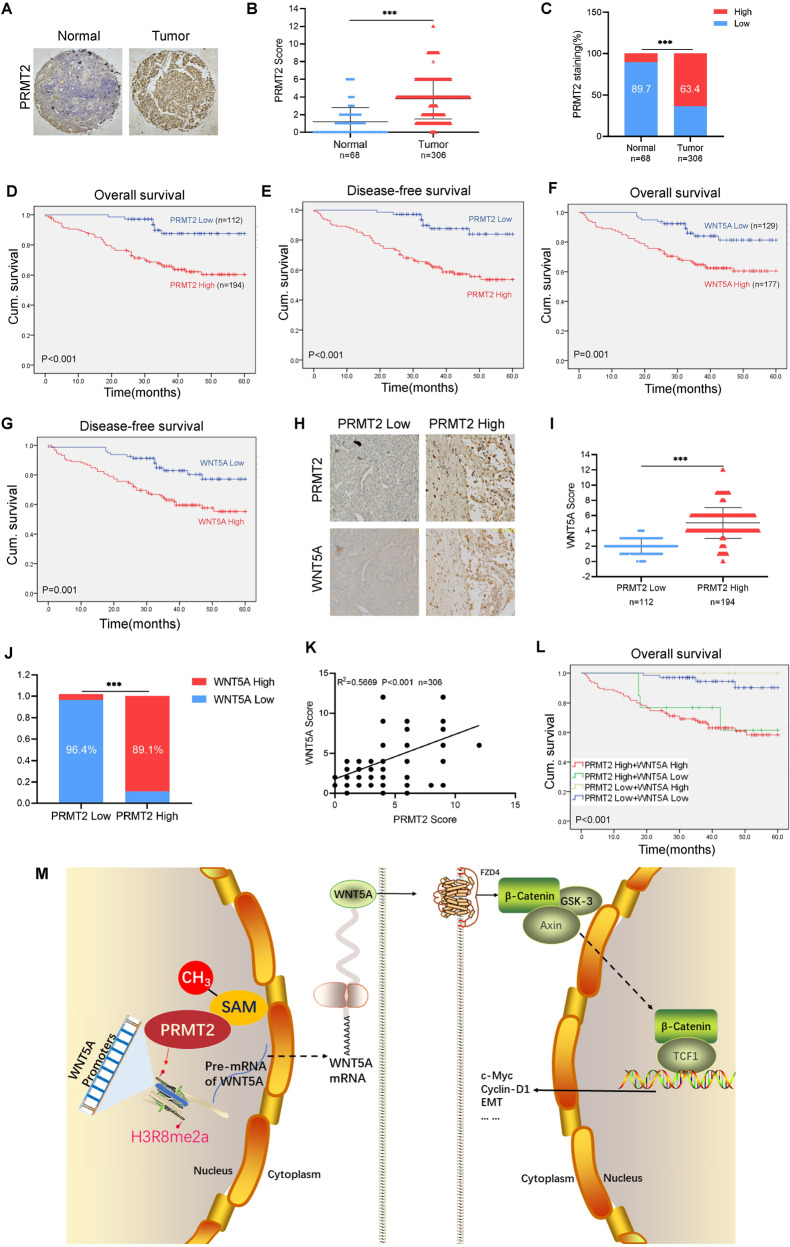
PRMT2 and WNT5A contribute to poor prognosis in RCC patients. **A**–**C** Representative images of PRMT2 IHC staining in normal renal tissue and renal cancer tissue from patients with RCC (**A**). IHC staining assays were performed with anti-PRMT2 and anti-WNT5A antibodies on tissue microarray (TMA) slides. Using the semiquantitative scoring method (using a scale from 0 to 12) for analyzing the scores of PRMT2 IHC staining (**B**, **C**). **D**, **E** High PRMT2 expression is correlated to poorer 5-year overall survival and disease-free survival for 306 patients with RCC (High PRMT2 expression patients 194, Low PRMT2 expression patients 112, test by log-rank test). **F**, **G** High WNT5A expression is correlated to poorer 5-year overall survival and disease-free survival for 306 patients with RCC (high WNT5A expression patients 177, low WNT5A expression patients 129, test by log-rank test). **H** Representative images of PRMT2 and WNT5A expressions in PRMT2-low case and PRMT2-high case were presented. **I**, **J** Correlation between PRMT2 and WNT5A expression were examined by chi-square exact test, respectively. **K** Correlation between PRMT2 and WNT5A expression were examined by Pearson correlation coefficient test, respectively. **L** High PRMT2 and WNT5A expression are correlated to poorer 5-year overall survival for 306 patients with RCC (high PRMT2 and high WNT5A expression patients 173, high PRMT2 and low WNT5A expression patients 21, low PRMT2 and high WNT5A expression patients 4, low PRMT2 and low WNT5A expression patients 108, test by log-rank test). **M** A proposed model for this study.

Finally, we evaluated our findings by assessing the clinical significance of PRMT2 and WNT5A expression in RCC patients. IHC staining assays were used to detect PRMT2 with an anti-PRMT2 antibody on tissue microarray (TMA) slides, which contained 68 normal kidney tissues and 306 RCC patient tissues. IHC staining showed that PRMT2 expression was sharply increased in RCC tissues compared to normal kidney tissues (Fig. 7A, B). Using Fisher’s exact test, we found that high PRMT2 expression in RCC tissues was approximately 63.4% (195/306) (Fig. 7C).

We then analyzed the correlation between PRMT2 expression and the clinicopathological characteristics of RCC patients. Our results showed that high PRMT2 expression was positively correlated with tumor size, depth of invasion, lymph node metastasis, distant metastasis, and TNM stage but not with age or sex (Supplementary Table 2). Moreover, we explored the correlation between PRMT2 expression and 5-year overall survival (OS) and 5-year disease-free survival (DFS) in patients with RCC using Kaplan–Meier survival analysis and log-rank tests. We found that patients with high PRMT2 expression had significantly worse 5-year OS and DFS outcomes than those with low PRMT2 expression (Fig. 7D, E and Supplementary Table 3). These data strongly suggest that high PRMT2 expression contributes to poor prognosis in patients with RCC.

Furthermore, we detected WNT5A expression by IHC staining assays in the TMA slides and analyzed the correlation between WNT5A expression and clinicopathological characteristics. The results revealed that high WNT5A expression was positively correlated with the depth of invasion, lymph node metastasis, distant metastasis, and TNM stage (Supplementary Table 4). We found that patients with high WNT5A expression had worse OS and DFS than those with low WNT5A expression (Fig. 7F, G and Supplementary Table 5). Our data indicate that WNT5A is positively correlated with malignant progression in RCC patients and might act as an independent risk factor for patients with RCC.

In addition, we analyzed the correlation between PRMT2 and WNT5A expression using IHC staining of RCC patient tissues (Fig. 7H). We found that high WNT5A expression was positively correlated with high PRMT2 expression (Fig. 7I, J). Consistently, we confirmed the positive correlation between PRMT2 and WNT5A expression using Pearson’s correlation analysis (Fig. 7K). Kaplan–Meier survival analysis and log-rank tests demonstrated that patients with high PRMT2 and WNT5A expression had the worst overall survival (Fig. 7L and Supplementary Table 6). Together, our data strongly suggest that high PRMT2 expression is strongly associated with high WNT5A expression, which significantly correlates with poor clinical outcomes in RCC patients.

## Discussion

Currently, effective therapeutic schedules targeting patients with metastatic RCC are lacking. Previous studies have shown that arginine methylation plays an important role in tumorigenesis and tumor development [33]. Here, we revealed that PRMT2 promotes RCC tumorigenesis and metastasis by enhancing WNT5A transcriptional expression. We demonstrated a positive correlation between PRMT2 and WNT5A expression in RCC patients. A schematic illustrating the proposed mechanism is shown in Fig. 7M: PRMT2-mediated H3R8me2a enrichment of the WNT5A promoter activates WNT5A transcription. Based on our findings, the repression of arginine methylation by targeting PRMT2 may be a promising therapeutic strategy for patient treatment.

In this study, we demonstrate that PRMT2 mediates H3R8me2a modification of the WNT5A promoter, which activates WNT5A transcription. In fact, H3R8me2a modification has been widely reported in various diseases [34]. H3R8me2a is a histone modification that regulates cancer progression by affecting the transcription of its target genes. Previous reports have shown that H3R8me2a modification can suppress the expression of several target genes [8]. However, an increasing number of recent studies have confirmed that H3R8me2a can facilitate the transcription of many target genes in several cancers [14, 32]. We propose that H3R8me2a has different regulatory models for target gene expression in various diseases.

It has been reported that WNT5A can activate noncanonical WNT signaling pathways by binding to a series of its receptors, such as frizzled (Fzd) or Ror family members [35]. Although WNT5A functions in RCC cells have not yet been clearly revealed, WNT5A signaling is crucial for regulating the proliferation and invasion of many other cancer cells [20]. Reports have shown that WNT5A has both oncogenic functions and tumor-suppressive functions in different cancers [35]. For instance, WNT5A suppresses cell growth and invasion in colon and thyroid cancer [20]. Studies have also shown that WNT5A can induce EMT and cell motility in lung cancer, prostate cancer, and melanoma [20]. Here, we demonstrated that PRMT2 activates WNT5A expression, which stimulates the Wnt signaling cascade and increases β-Catenin, c-Myc, and Snail-1 expression. We found that WNT5A enhanced the proliferation and motility of RCC cells. Therefore, our study strongly indicates that WNT5A has oncogenic functions in RCC cells (promoting RCC cell proliferation, EMT, and migration).

In the last 10 years, a lot of studies have demonstrated that members of the PRMT family play critical roles in cancer progression. The development of small-molecule inhibitors targeting PRMTs is a promising cancer therapeutic strategy. For instance, the PRMT5-specific inhibitor GSK3326595 has entered into phase II clinical trials for cancer therapy [8]. Herein, we showed that PRMT2 facilitates RCC cancer progression by regulating H3R8me2a modification on the WNT5A promoter. To date, no specific inhibitors of PRMT2 have been reported, but we believe that developing specific inhibitors targeting PRMT2 is a promising clinical treatment strategy for RCC patients.

In conclusion, our study showed that PRMT2 is a potential biomarker for the diagnosis and evaluation of metastasis in RCC patients. We believe that targeting PRMT2 may be a promising therapeutic strategy for RCC patients.

## Supplementary information

Supplementary Information

Figure S1

Figure S2

Figure S3

Figure S4

Supplementary Table 1

Supplementary Table 2

Supplementary Table 3

Supplementary Table 4

Supplementary Table 5

Supplementary Table 6

Supplementary Table 7

aj-checklist

original data of uncropped WB images

## Data Availability

Data supporting the findings of this study are available from the corresponding author upon reasonable request.
