# Supplementary material for: PRMT2 promotes RCC tumorigenesis and metastasis via enhancing WNT5A transcriptional expression
Source: Cell Death Dis. 2023 May 12;14(5):322. doi: 10.1038/s41419-023-05837-6 (PMC10182089; doi:10.1038/s41419-023-05837-6)
Supplement: Supplementary file 1 — Supplementary Information [file 41419_2023_5837_MOESM1_ESM.docx]

**PRMT2 promotes RCC tumorigenesis and metastasis via enhancing WNT5A transcriptional expression**

Zhongwei Li^#1,2,3^, Chaozhen Chen^#4,1^, Hongmei Yong^#5^, Lei Jiang^4,1^, Pengfei Wang^1^, Sen Meng^1^, Sufang Chu^1^, Zhen Li^4,1^, Qingxiang Guo^4,1^, Junnian Zheng*^1,2,3^, Jin Bai*^1,2,3^ and Hailong Li*^1,4^

^1^Cancer Institute, Xuzhou Medical University, Xuzhou, Jiangsu, China.

^2^Center of Clinical Oncology, the Affiliated Hospital of Xuzhou Medical University, Xuzhou, Jiangsu, China.

^3^Jiangsu Center for the Collaboration and Innovation of Cancer Biotherapy, Cancer Institute, Xuzhou Medical University, Xuzhou, Jiangsu, China.

^4^Department of Urology, The Affiliated Hospital of Xuzhou Medical University, Xuzhou, Jiangsu, China.

^5^Department of Oncology, The Affiliated Huai'an Hospital of Xuzhou Medical University and The Second People's Hospital of Huai'an, Huaian, Jiangsu, China

**Authorship notes:** #These authors contributed equally to this work.

**Running title**: PRMT2 facilitates RCC malignancy through WNT5A.

***Correspondence Authors:**

Hailong Li, Cancer Institute, Xuzhou Medical University. 209 Tongshan Road, Xuzhou, 221004, Jiangsu Province, China. E-mail:justinlee719@hotmail.com.

Jin Bai,Cancer Institute, Xuzhou Medical University. 209 Tongshan Road, Xuzhou, 221004, Jiangsu Province, China. E-mail: bj@xzhmu.edu.cn.

Junnian Zheng, Cancer Institute, Xuzhou Medical University. 209 Tongshan Road, Xuzhou, 221004, Jiangsu Province, China. E-mail: jnzheng@xzhmu.edu.cn.

**Conflict of interest:** No potential conflicts of interest were disclosed by the authors.

**Supplementary Materials**

**1. The sequences of shRNAs using for gene silencing**

|  | 5’ | stem | loop | stem | 3’ |
| --- | --- | --- | --- | --- | --- |
| shPRMT2#1-F | CCGG | GAACGGCTTTGCTGACATCAT | CTCGAG | ATGATGTCAGCAAAGCCGTTC | TTTTTG |
| shPRMT2#1-R | AATTCAAAAA | GAACGGCTTTGCTGACATCAT | CTCGAG | ATGATGTCAGCAAAGCCGTTC |  |
| shPRMT2#2-F | CCGG | GACTGGGATCATCAGTCTCTT | CTCGAG | AAGAGACTGATGATCCCAGTC | TTTTTG |
| shPRMT2#2-R | AATTCAAAAA | GACTGGGATCATCAGTCTCTT | CTCGAG | AAGAGACTGATGATCCCAGTC |  |

**2. The primers using for quantitative RT-PCR**

| Primer Name | Sequence 5’-3’ |
| --- | --- |
| GAPDH(forward) | GCACCGTCAAGGCTGAGAAC |
| GAPDH(reverse) | TGGTGAAGACGCCAGTGGA |
| PRMT2(forward) | GCAGTTGGACATGAGAACCGTG |
| PRMT2(reverse) | AGGCTCTGGAAGTGGACGCTAA |
| WNT2B(forward) | TGGATGCCAAGGAGAAGAGGCT |
| WNT2B(reverse) | GTACAGGAACCACTCACGCCAT |
| WNT5B(forward) | CAAGGAATGCCAGCACCAGTTC |
| WNT5B(reverse) | CGGCTGATGGCGTTGACCACG |
| WNT5A(forward) | TACGAGAGTGCTCGCATCCTCA |
| WNT5A(reverse) | TGTCTTCAGGCTACATGAGCCG |
| ChIP-1(forward） | AACAACTCTTTATCTGAAGTCCTTGGG |
| ChIP-1(reverse) | ACATGACAAGACATAATCTTGATTTATTCC |
| ChIP-2(forward) | GAGGTCCAGTTTTACCACCACAGG |
| ChIP-2(reverse) | TTCTTCCTCCCGATGTCCGTCT |
| ChIP-3(forward） | TCATGAAATTTCTGCTTGCTGCTGT |
| ChIP-3(reverse) | TATTTCCTGTTCTGCAGTCTTTCCTCT |
| ChIP-4(forward） | GCACTGTTTGGGCTGTGTCTATACA |
| ChIP-4(reverse) | CAGTTCGTGTAGAGGATCCTATTCTATGA |
| ChIP-5(forward） | CCTTGGACTCTGGGACACTGG |
| ChIP-5(reverse) | GAGATTCCCTGGCTGCGG |

**3. The sequence of WNT5A promoter region (-1000--0bp)**

GATAAAACAACTCTTTATCTGAAGTCCTTGGGACTAGACGGTTTTGTTGGAAATTTTGGGAAAATAAAATGGGGCATATTGCTATTAATTTGCAAGCAGCCCAGTGAGGTCTGGGGAAATACCCAGTAACCAAATGCATTTGTATTCATTCAGTGAAGAGTATTCCTAGCAAGTGGAATAAATCAAGATTATGTCTTGTCATGTGAGGTCCAGTTTTACCACCACAGGAGTTTGAAAACCTCTGTTTTCAGAGCTGCTTTTTTTCTAGAAGAGCAGGCATTGAGTGCCAGGTAAGGGCCTGACCCATGCTAAGAGCATTCCTAGATATCGTGTTTCATTCTCAGTACTGCCCTGATTTTCAAGCTCATCTCATTTCATTCTCAATGCATTTCTGCAAGGTAGATCATGAAATTTCTGCTTGCTGCTGTTGTTGCTGTTGTTGTGTGTAAGAGGGTTCTGAAGTGCTGGCTGAGAGGCTAGACAAGGTGTGACAGCTGGAAAGTGGCAGGGCAGGGACTGGAACTCCAGATTCCACAGCTGGCACTCTTAACCACTAACCAACACCGACTTCCACTAGAGGAAAGACTGCAGAACAGGAAATAGCACTGTTTGGGCTGTGTCTATACAAGCAAAGGCAATTGGAGAGAAGCAGGTGATTATCCAGATAACTCAGAAAATAGCCATTAAAAAATAAAGGTTTGTGGTTGGGTAGCCAGTATTTCTAGACTGGCTGTATGTGTGGAGGAAATGCCCAGGTTAACGGCCCTTTTCTTCATAGAATAGGATCCTCTACACGAACTGCCTTGGACTCTGGGACACTGGGATGCTAGTTTCTCCATTCTTCGGGAGCGCCCTTTGGCAGCCCCTTTCATAGTCAGCTCATATCCTGCCACTTCTTGGAGCTGCTGCACGTAAGGTGGGTCCCGGAGATCCCTGCCCGCGGGGCGGGGCTGGAGTGTCTGCCTAGGAGGCACATGCTCTCCCGCAGCCAGGGAATCTC

**Supplementary Methods**

**1. RNA extract, reverse transcription, and qRT-PCR**

Total RNA was extracted from cells using the Trizol reagent (Invitrogen) following manufacturer's instructions. The cDNA was generated with the HiScript Q RT SuperMix for qPCR (Vazyme). Real-Time PCR was carried out on a Roche LightCycler480 using SYBR Green Realtime PCR Master Mix (TOYOBO, Osaka, Japan).PCR reactions were run in triplicate for three independent experiments.

**2. RNA extract, reverse transcription, and qRT-PCR**

Cells were washed twice in cold PBS and lysed in RIPA lysis buffer (50mM Tris-HCl, pH 7.4, 150mM NaCl, 1% sodium deoxycholate, 1%Triton X-100 and 0.1% SDS) plus protease inhibitor cocktail (Roche, Mannheim, Germany). Protein lysates were subjected to SDS-PAGE, transferred to NC membrane (Pall Corporation, USA) and detected with appropriate primary antibodies coupled with HRP-conjugated secondary antibodies by ECL reagent (GE Healthcare, Buckinghamshire, UK).

**3. Cell proliferation assays**

Cell growth rates were assessed by the CCK-8 assay. Cells were seeded onto 96-well plates in triplicate. After incubation for indicated time, 20μlWST–8 and 200μl empty medium were added to each well and incubated for 1h at 37 °C before removal of the culture medium. Cell viability was determined by measuring the absorbance at 450 nm. The experiments were performed at least three times.

**4. Transwell migration and invasion assays**

In vitro cell migration and invasion assays were performed using transwell chambers with polyethylene terephthalate membrane (24-well inserts, 12.0μm; Corning). For the migration assay, 2×10^4^ cells were added to the top chambers. For the invasion assay, 2×10^4^ cells were seeded into the top chamber coated with Matrigel (BD Biosciences). Complete medium was added to the bottom wells to stimulate migration or invasion. After cells were incubated for 24-48h, they were stained with 0.1% Crystal Violet. Five fields per filter were counted.

**5. Cell cycle analysis**

Forty-eight hours after transfection, the cells were synchronized by serum starvation overnight and induced re-enter cell cycle by incubating in medium containing 10% fetal bovine serum for 4 h. The detection was applied with Cell cycle Assay Kit (KGA512, Keygen).The cells were collected, washed by PBS, and fixed in pro-cooled 70% ethanol at 4 °C overnight. The day after, the cells were washed twice with PBS and resuspended in RNase A at 37 °C for 30 min, and then propidium iodide (PI) was added to the cells in the dark at 4 °C for 30 min. In the end, all samples were analyzed by flow cytometry (BD, FACS CantoTM II).

**6. Luciferase reporter assay**

Dual-luciferase reporter assays were carried out using the Dual-luciferase Reporter Assay System (Promega GloMax, USA). We constructed reporter plasmid with pGL3.0-Basic bearing WNT5A promoter regions. Next, we co-transfected reporter plasmid and Renilla into preprocessed cells. Cells were then collected and lysed for luciferase detection 48 h after transfection. The relative luciferase activity was normalized against to the Renilla luciferase activity.

**7. Chromatin immunoprecipitation (ChIP) assay**

ChIP assay was performed using the ChIP Assay Kit (Beyotime Biotechnology, P2078) according to the manufacturer’s protocol.

**8. Stable cell lines generation**

First lentiviruses were generated by co-transfecting HEK293T cells with shPRMT2#1 or control shRNA with the packaging plasmids (pMD2G and psPAX; GenePharma, Shanghai, China). Virus-containing supernatant was harvested 48h post-transfection. ACHN cell line was infected with the virus to create stable cell lines and purinomycin was used for screening 48 hours after infection.

**9. Animal works of tumour xenograft model and lung-colonisation metastasis model**

BALB/c nude mice (6–8 weeks old) were purchased from Beijing Vital River Laboratory Animal Technology Co., Ltd. (Beijing, China). All animal experiments were approved by the Animal Care and Use Committee at Xuzhou Medical University. For subcutaneous tumor model, ACHN-Vector and ACHN-shPRMT2#1 cells (1×10^7^) were inoculated ventrally onto the mice. The mice were sacrificed after four weeks, and the subcutaneous tumors were weighed. For lung metastasis model, ACHN-vector and ACHN-shPRMT2#1 cells (5×10^6^) were injected into the mice through the caudal vein. Mice were sacrificed after 8 weeks, and lung metastatic nodules were examined after fixation using Bouin’s method.

**10. Patient specimens and tissue microarray construction**

Tissue microarray (TMA) slides including 68 normal tissues and 306 RCC tissues which were enrolled at Affiliated Hospital of Xuzhou Medical University from 2005 to 2008 in China. Five-year clinical follow-up results were available for 306 patients. The use of these tissue specimens was approved by the Ethics Committee of the Hospital. Also, these specimens and patient data were de-identified prior to use in our study.

**11. Immunohistochemistry (IHC)**

Heat-induced epitope retrieval was performed with retrieval buffer (citrate buffer pH 6.0) before the IHC staining protocol. Slides were incubated with anti-PRMT2 antibody(1:100) or anti-WNT5A antibody(1:100) at 4^o^C overnight. The resulting slides were treated with 3, 3′-diaminobenzidine to produce a brown precipitate. Non-immune serum was used as negative control.

**12. Assessment of IHC**

Three pathologists assessed separately the TMAs under blinded experimental conditions and all differences that arise were resolved by discussion. The staining scores of PRMT2 and WNT5A were evaluated via combining the percentage of cells with the staining intensity and being dependent on the IRS (immunoreactivity score, IRS). The intensity of PRMT2 and WNT5A immunostaining were scored as 0–3 (0, negative; 1, weak; 2, moderate; 3, strong); the percentage of immunoreactivity cells was graded as 1 (0–25%), 2 (26–50%), 3 (51–75%), and 4 (76–100%). Relied on the IRS, the level of PRMT2 and WNT5A expression was categorized as low (IRS: 0–3) and high (IRS: 4–12) expression.

**Supplementary Figure Legends**

**Supplementary Figure 1. A** Detection of PRMT2 knockdown efficiency in RCC cell lines by qRT-PCR. **B** Detection of β-catenin and c-Myc expression after overexpression of PRMT2 in HEK293T cells.

**Supplementary Figure 2. A** Detection of myc-PRMT2 overexpression by western blot in HK-2 cells. **B** CCK8 assays detection of HK-2 cells proliferation ability after over-expressing PRMT2 in HK-2 cells. **C** Transwell assays detection of HK-2 cells motility after over-expressing PRMT2 in HK-2 cells.

**Supplementary Figure 3. A-C** The ChIP primers binding with WNT5A promoter was detected by qRT-PCR assays after ChIP assays using anti-H3R8me2a.

**Supplementary Figure 4. A, B** CCK8 assays detection of RCC cells proliferation ability after treated with LiCl in knockdown PRMT2 cells. **C,D** Transwell assays detection of RCC cells motility after treated with LiCl in knockdown PRMT2 cells.
