# Supplementary figures and images for: PRMT2 promotes RCC tumorigenesis and metastasis via enhancing WNT5A transcriptional expression
Source: Cell Death Dis. 2023 May 12;14(5):322. doi: 10.1038/s41419-023-05837-6 (PMC10182089; doi:10.1038/s41419-023-05837-6)

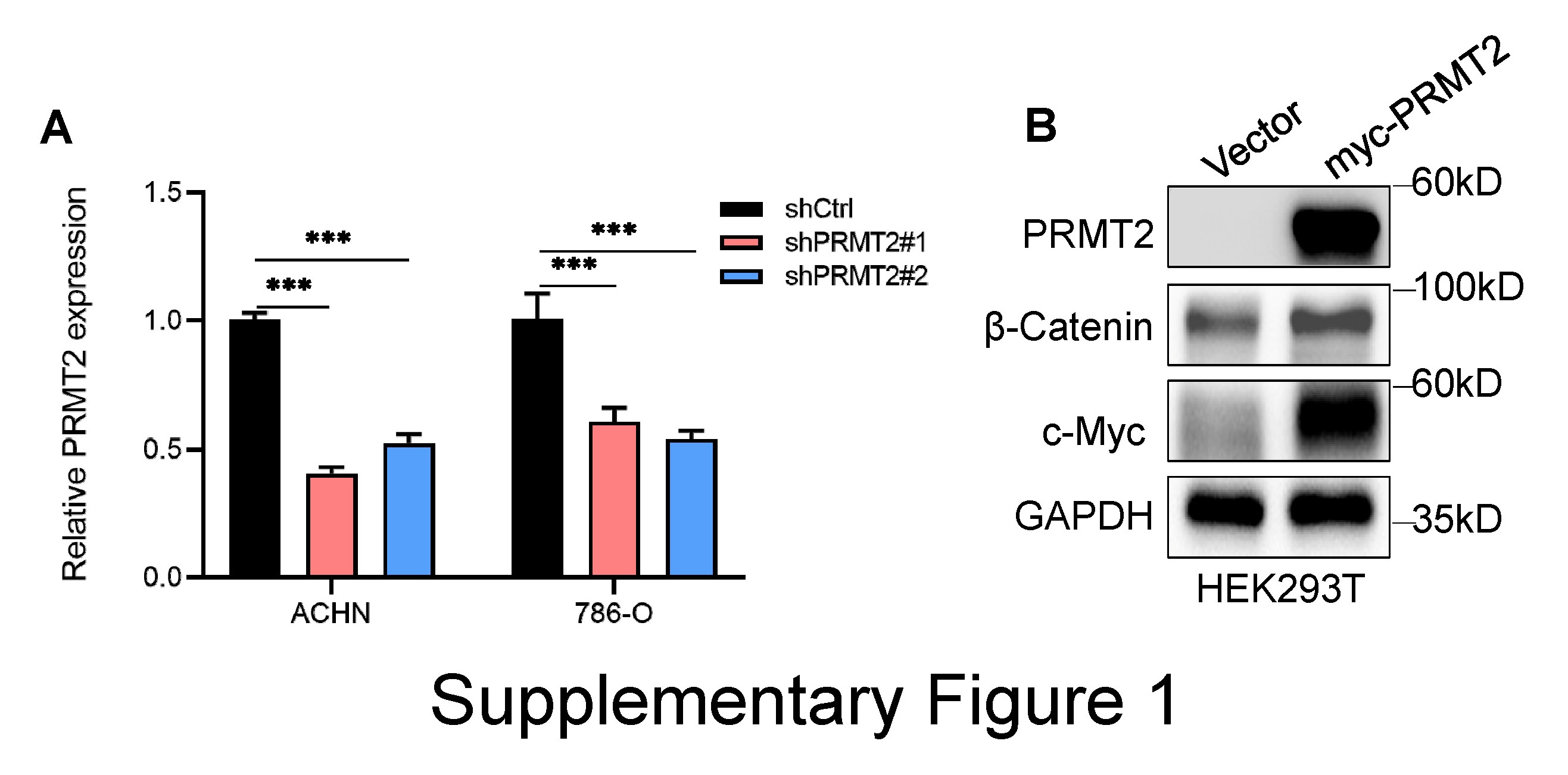

Supplement: Supplementary file 2 — Figure S1 [file 41419_2023_5837_MOESM2_ESM.jpg]

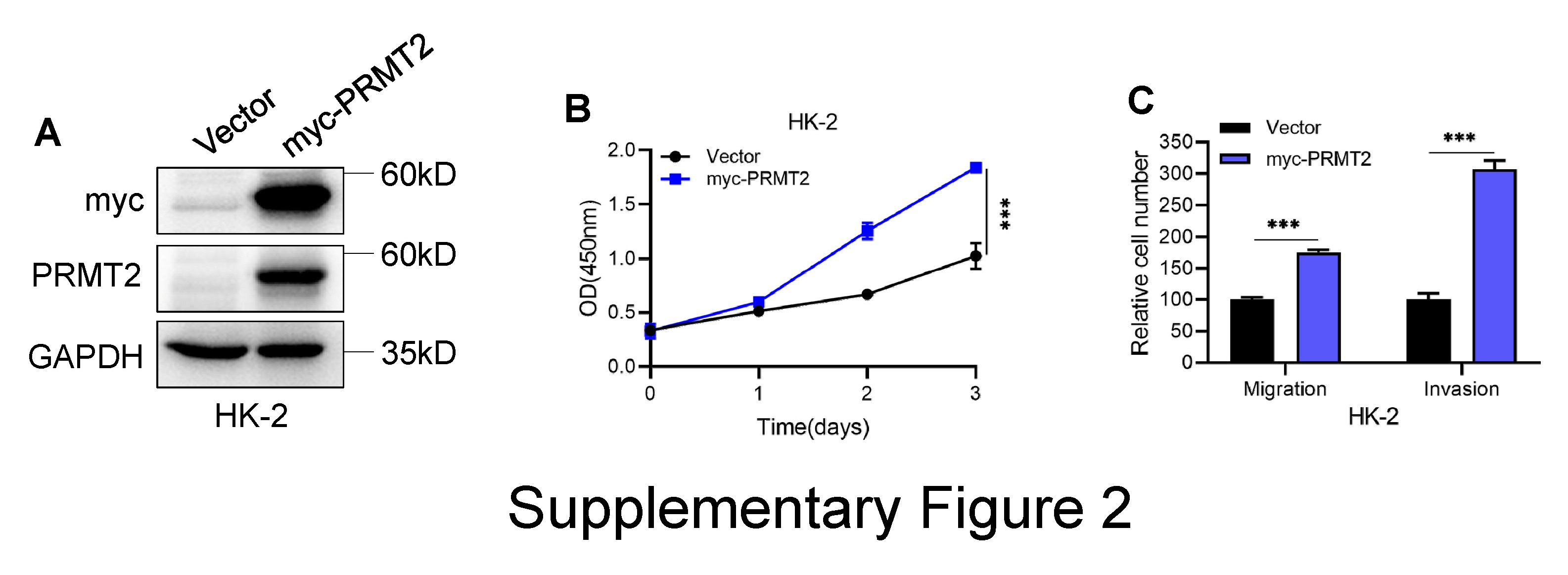

Supplement: Supplementary file 3 — Figure S2 [file 41419_2023_5837_MOESM3_ESM.jpg]

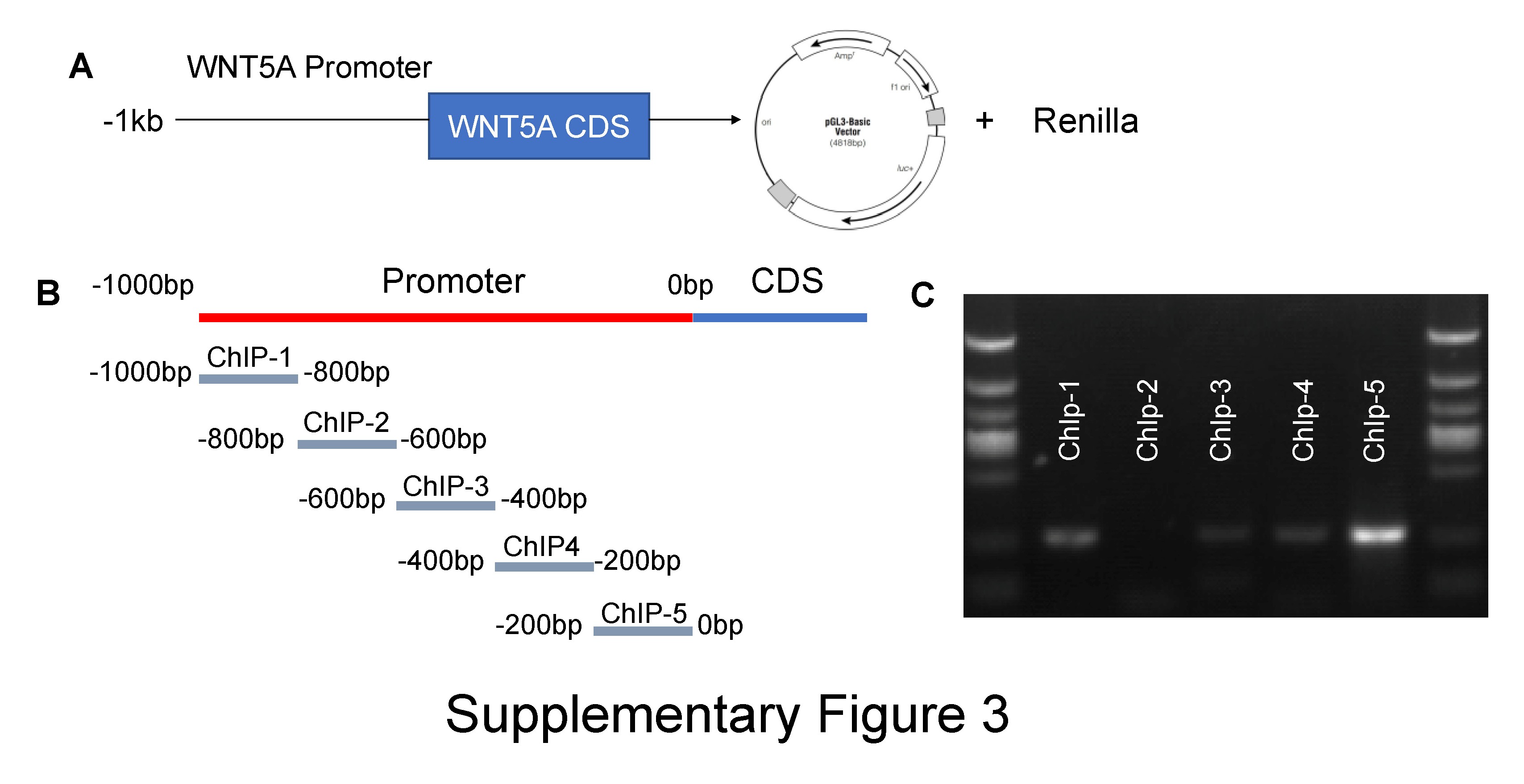

Supplement: Supplementary file 4 — Figure S3 [file 41419_2023_5837_MOESM4_ESM.jpg]

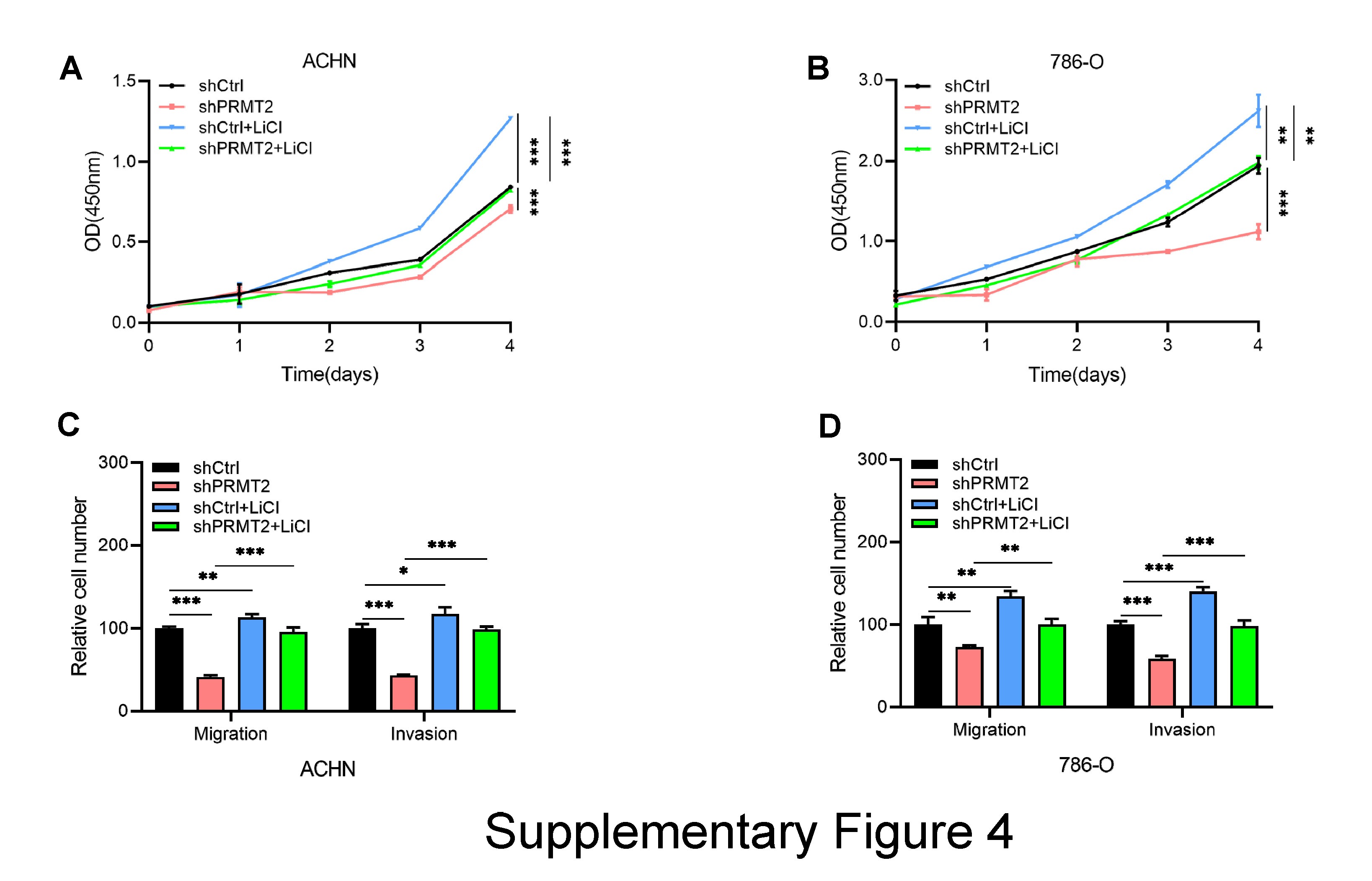

Supplement: Supplementary file 5 — Figure S4 [file 41419_2023_5837_MOESM5_ESM.jpg]

fig 1

F

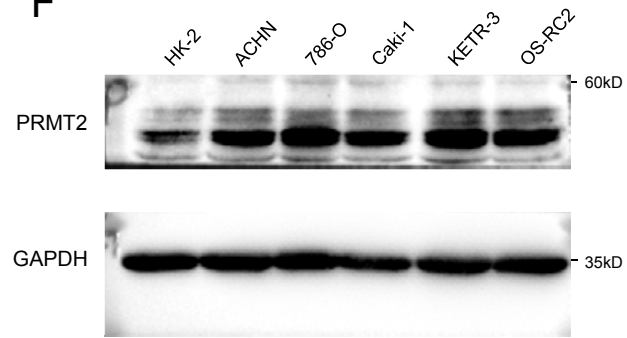



fig 3

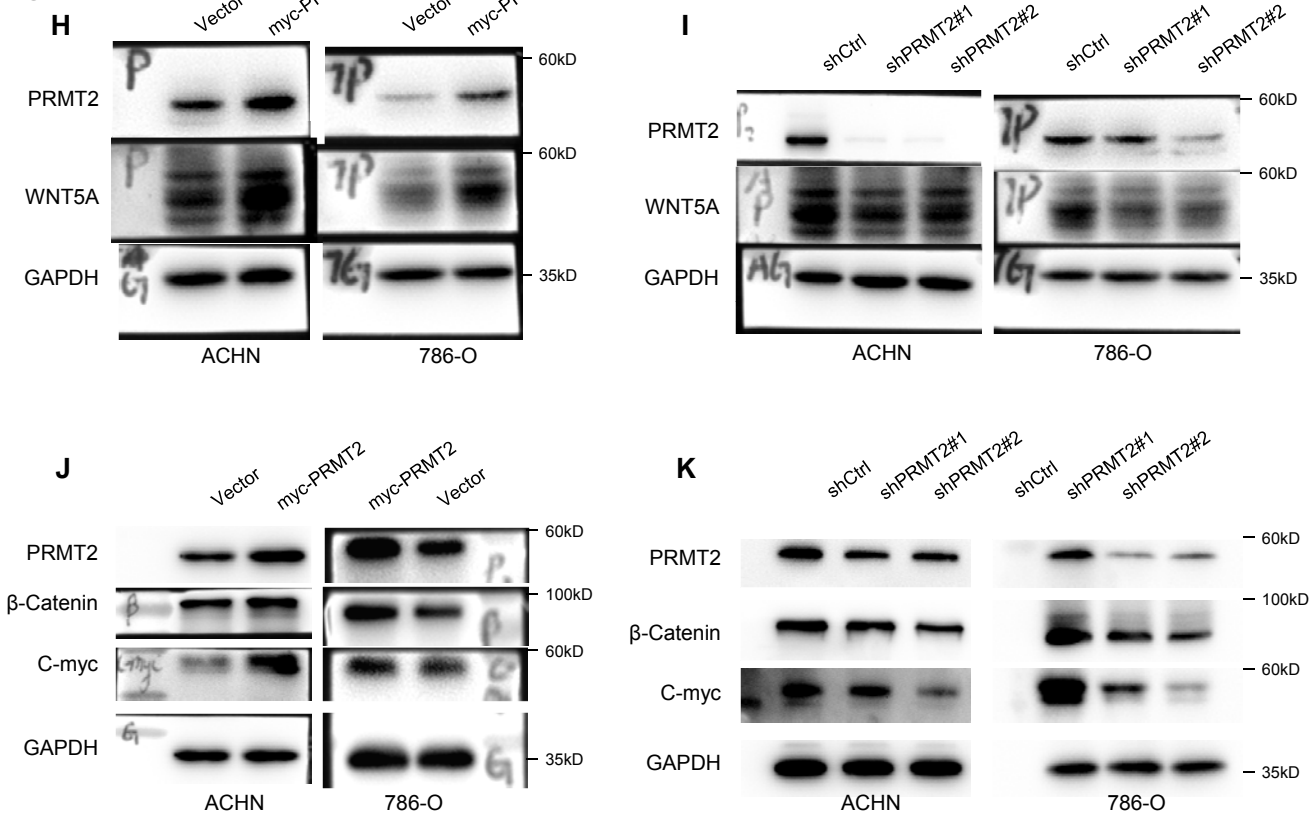

fig 4

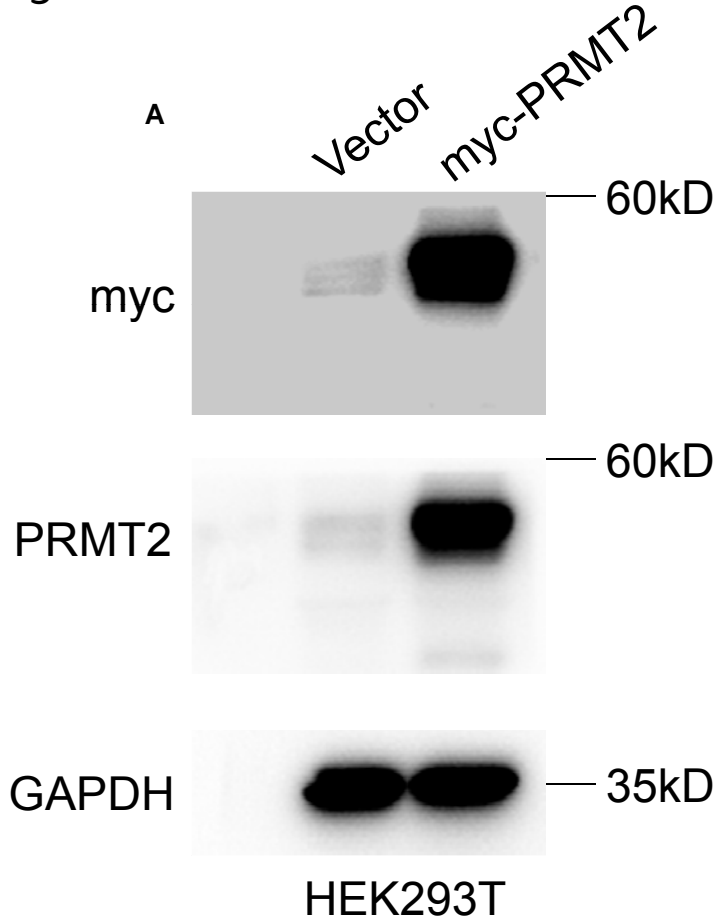

fig 5

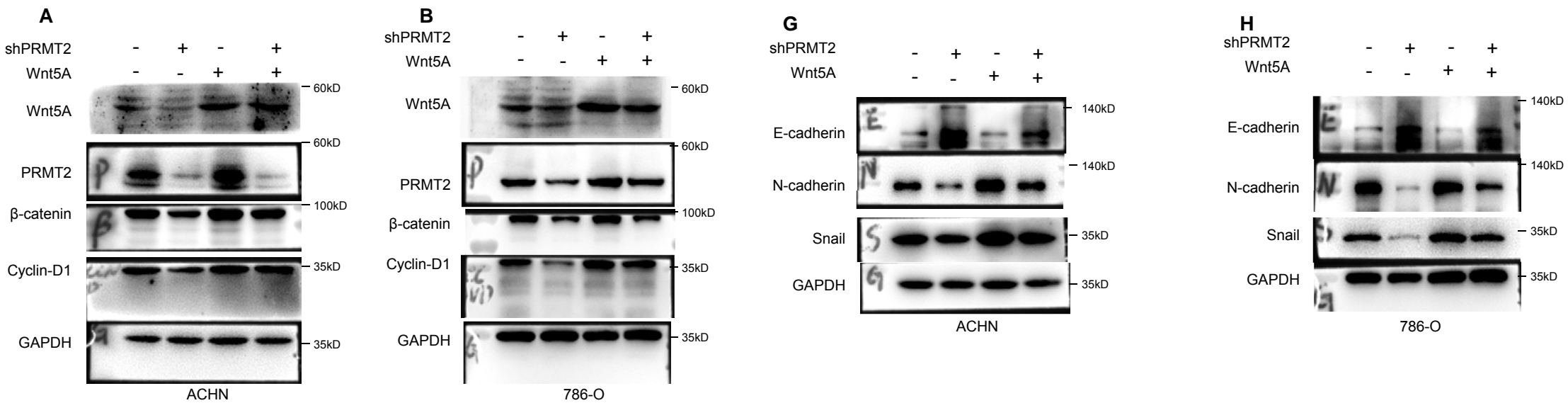

Supplementary Figure 1

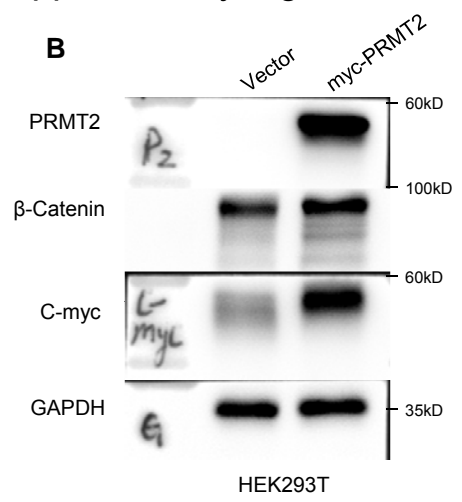

Supplementary Figure 2

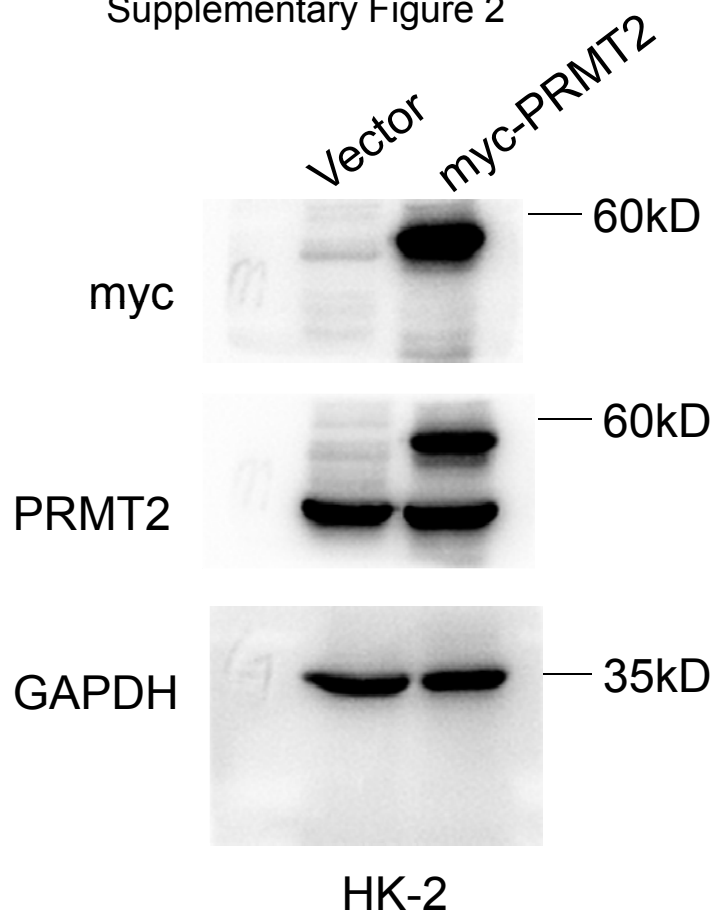

Supplementary Figure 3

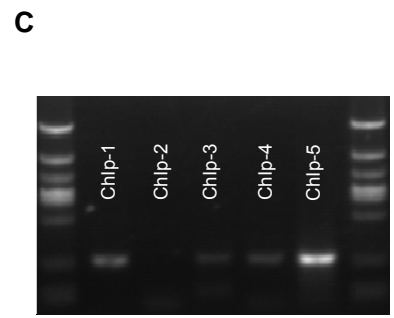

Supplement: Supplementary file 14 — original data of uncropped WB images [file 41419_2023_5837_MOESM14_ESM.pdf]
