# Supplementary material for: PRMT2 promotes RCC tumorigenesis and metastasis via enhancing WNT5A transcriptional expression
Source: Cell Death Dis. 2023 May 12;14(5):322. doi: 10.1038/s41419-023-05837-6 (PMC10182089; doi:10.1038/s41419-023-05837-6)
Supplement: Supplementary file 7 — Supplementary Table 2 [file 41419_2023_5837_MOESM7_ESM.docx]

**Supplementary Table 2** PRMT2 staining and clinicopathological characteristics of 306 cancer patients.

| **Variables** | **PRMT2 staining** | | | |
| --- | --- | --- | --- | --- |
|  | **Low (%)** | **High (%)** | **Total** | ***P* *** |
| **Age** |  |  |  |  |
| ≤56 | 58(39.4) | 89(60.6) | 147 | 0.266 |
| >56 | 53(33.3) | 106(66.7) | 159 |  |
| **Gender** |  |  |  |  |
| Male | 80(39.4) | 123(60.6) | 203 | 0.109 |
| Female | 31(30.0) | 72(70.0) | 103 |  |
| **Tumor size** |  |  |  |  |
| T1(≤7cm) | 94(39.7) | 143(60.3) | 237 | 0.022 |
| T2(>7cm) | 17(24.6) | 52(75.4) | 69 |  |
| **Depth of invasion** |  |  |  |  |
| Intrarenal | 88(43.8) | 113(56.2) | 201 | <0.001 |
| Extrarenal | 23(21.9) | 82(78.1) | 105 |  |
| **Lymph node metastasis** |  |  |  |  |
| Negative | 110(99.1) | 1(0.9) | 111 | 0.001 |
| Positive | 170(87.1) | 25(12.9) | 195 |  |
| **Distant metastasis** |  |  |  |  |
| Negative | 111(42.2) | 170(57.8) | 263 | <0.001 |
| Positive | 0(0) | 43(100) | 43 |  |
| **TNM stage** |  |  |  |  |
| T1/T2 | 99(44.8) | 122(55.2) | 221 | <0.001 |
| T3/T4 | 12(14.1) | 73(85.9) | 85 |  |
| **Urinary system diseases** |  |  |  |  |
| Negative | 101(35.3) | 185(64.7) | 286 | 0.140 |
| Positive | 10(50) | 10(50) | 20 |  |

^*^*P* values are from χ^2^ test.
