# Supplementary material for: PRMT2 promotes RCC tumorigenesis and metastasis via enhancing WNT5A transcriptional expression
Source: Cell Death Dis. 2023 May 12;14(5):322. doi: 10.1038/s41419-023-05837-6 (PMC10182089; doi:10.1038/s41419-023-05837-6)
Supplement: Supplementary file 8 — Supplementary Table 3 [file 41419_2023_5837_MOESM8_ESM.docx]

**Supplementary Table 3** Multivariate Cox regression analysis PRMT2 on 5-year overall survival of 306 renal cancer patients.

| Variable^*^ | Overall survival | | | |  | |
| --- | --- | --- | --- | --- | --- | --- |
|  | Hazard ratio | | 95% CI^†^ | *P* |  | |
| PRMT2 | 3.366 | 1.456-7.784 | | 0.005 |  | |
| Age | 0.917 | 0.530-1.586 | | 0.758 |  | |
| Gender | 0.888 | 0.485-1.623 | | 0.698 |  | |
| Tumor size | 3.118 | 1.718-5.659 | | 0.000 |  | |
| Depth of invasion | 2.385 | 1.774-3.207 | | <0.001 | |  |
| Distant metastasis | 1.412 | 1.007-1.975 | | 0.046 |  | |
|  |  |  | |  |  | |

^*^Coding of variables: PRMT2 was coded as 1 (low), and 2 (high). Gender was coded as 1 (male), and 2 (female). Tumor size was coded as 1 (≤7cm), and 2 (>7 cm). Depth of invasion was coded as 1 (intra-renal), and 2 (extra-renal).Distance metastasis was coded as 1 (negative), and 2 (positive).

^†^ CI: confidence interval.
