# Supplementary material for: PRMT2 promotes RCC tumorigenesis and metastasis via enhancing WNT5A transcriptional expression
Source: Cell Death Dis. 2023 May 12;14(5):322. doi: 10.1038/s41419-023-05837-6 (PMC10182089; doi:10.1038/s41419-023-05837-6)
Supplement: Supplementary file 9 — Supplementary Table 4 [file 41419_2023_5837_MOESM9_ESM.docx]

**Supplementary Table 4** WN5A staining and clinicopathological characteristics of 306 renal cancer patients.

| **Variables** | **WNT5A staining** | | | | |
| --- | --- | --- | --- | --- | --- |
|  | **Low (%)** | **High (%)** | **Total** | | ***P* *** |
| **Age** |  |  |  |  | |
| ≤56 | 63(42.8) | 84(57.2) | 147 | 0.899 | |
| >56 | 67(42.1) | 92(57.9) | 159 |  | |
| **Gender** |  |  |  |  | |
| Male | 92(45.3) | 111(54.7) | 203 | 0.116 | |
| Female | 37(35.9) | 66(64.1) | 103 |  | |
| **Tumor size** |  |  |  |  | |
| T1(≤7cm) | 105(44.1) | 133(55.9) | 238 | 0.279 | |
| T2(>7cm) | 25(36.8) | 43(63.2) | 68 |  | |
| **Depth of invasion** |  |  |  |  | |
| Intrarenal | 116(46.8) | 132(53.2) | 248 | 0.002 | |
| Extrarenal | 14(24.1) | 44(75.9) | 58 |  | |
| **Lymph node metastasis** |  |  |  |  | |
| Negative | 125(44.6) | 155(55.4) | 280 | 0.021 | |
| Positive | 5(19.2) | 21(80.8) | 26 |  | |
| **Distant metastasis** |  |  |  |  | |
| Negative | 117(45.5) | 140(54.5) | 257 | 0.014 | |
| Positive | 13(26.5) | 36(73.5) | 49 |  | |
| **TNM stage** |  |  |  |  | |
| T1/T2 | 109(49.5) | 111(50.5) | 220 | <0.001 | |
| T3/T4 | 21(24.4) | 65(75.6) | 86 |  | |
| **Urinary system diseases** |  |  |  |  | |
| Negative | 120(41.8) | 167(58.2) | 287 | 0.355 | |
| Positive | 10(52.6) | 9(47.7) | 19 |  | |

^*^*P* values are from χ^2^ test.
