# Supplementary material for: PRMT2 promotes RCC tumorigenesis and metastasis via enhancing WNT5A transcriptional expression
Source: Cell Death Dis. 2023 May 12;14(5):322. doi: 10.1038/s41419-023-05837-6 (PMC10182089; doi:10.1038/s41419-023-05837-6)
Supplement: Supplementary file 10 — Supplementary Table 5 [file 41419_2023_5837_MOESM10_ESM.docx]

**Supplementary Table 5** Multivariate Cox regression analysis WNT5A on 5-year overall survival of 306 renal cancer patients.

| Variable^*^ | Overall survival | | | |  |
| --- | --- | --- | --- | --- | --- |
|  | Hazard ratio | | 95% CI^†^ | *P* |  |
| WNT5A | 2.831 | 1.329-6.029 | | 0.007 |  |
| Age | 1.570 | 0.877-2.810 | | 0.129 |  |
| Gender | 1.083 | 0.783-1.522 | | 0.791 |  |
| Tumor size | 2.403 | 1.211-4.768 | | 0.012 |  |
| Depth of invasion | 1.566 | 0.776-3.159 | | 0.210 |  |
| Distant metastasis | 2.432 | 1.252-4.727 | | 0.009 |  |

^*^Coding of variables: WNT5A was coded as 1 (low), and 2 (high). Gender was coded as 1 (male), and 2 (female). Tumor size was coded as 1 (≤7cm), and 2 (>7 cm). Depth of invasion was coded as 1 (intra-renal), and 2 (extra-renal).Distance metastasis was coded as 1 (negative), and 2 (positive).

^†^ CI: confidence interval.
