# Supplementary material for: PRMT2 promotes RCC tumorigenesis and metastasis via enhancing WNT5A transcriptional expression
Source: Cell Death Dis. 2023 May 12;14(5):322. doi: 10.1038/s41419-023-05837-6 (PMC10182089; doi:10.1038/s41419-023-05837-6)
Supplement: Supplementary file 11 — Supplementary Table 6 [file 41419_2023_5837_MOESM11_ESM.docx]

**Supplementary Table 6** Univariate Cox proportional regression analysis PRMT2 and WNT5A on 5-year overall survival of 306 renal cancer patients.

| Variable^*^ | Overall survival | | |
| --- | --- | --- | --- |
|  | Hazard ratio | 95% CI^†^ | *P*^*^ |
| **PRMT2** |  |  |  |
| Low | 1.000 |  |  |
| High | 4.142 | 1.441-11.905 | 0.008 |
| **WNT5A** |  |  |  |
| Low | 1.000 |  |  |
| High | 3.430 | 1.666-7.060 | 0.001 |
| **Age** |  |  |  |
| ≤56 years | 1.000 |  |  |
| >56 years | 1.508 | 0.856-2.656 | 0.155 |
| **Gender** |  |  |  |
| Male | 1.000 |  |  |
| Female | 1.194 | 0.678-2.103 | 0.539 |
| **Tumor size** |  |  |  |
| ≤7 cm | 1.000 |  |  |
| >7 cm | 4.145 | 2.360-7.281 | <0.001 |
| **Depth of invasion** |  |  |  |
| Intra-renal | 1.000 |  |  |
| Extra-renal | 3.379 | 1.921-5.941 | <0.001 |
| **Distant metastasis** |  |  |  |
| Negative | 1.000 |  |  |
| positive | 4.109 | 2.347-7.195 | <0.001 |
| **TNM stage** |  |  |  |
| T1/T2 | 1.000 |  |  |
| T3/T4 | 7.236 | 3.837-13.649 | <0.001 |
| **Urinary system diseases** |  |  |  |
| Negative | 1.000 |  |  |
| positive | 0.876 | 0.454-1.666 | 0.674 |

* *P* values are from Log-rank test.† CI: confidence interval.
